# Persistence of pharmacological treatment into adulthood, in UK primary care, for ADHD patients who started treatment in childhood or adolescence

**DOI:** 10.1186/1471-244X-12-219

**Published:** 2012-12-05

**Authors:** Suzanne McCarthy, Lynda Wilton, Macey L Murray, Paul Hodgkins, Philip Asherson, Ian CK Wong

**Affiliations:** 1 School of Pharmacy, University College Cork, Cork, Ireland; 2 Pharmacy Department, Cork University Hospital, Cork, Ireland; 3 Centre for Paediatric Pharmacy Research, UCL School of Pharmacy, London, UK; 4 Shire Pharmaceuticals LLC, Wayne, PA, USA; 5 MRC Social, Genetic and Developmental Psychiatry Centre, Institute of Psychiatry, London, UK; 6 Centre for Safe Medication Practice and Research, Department of Pharmacology and Pharmacy, Li Ka Shing Faculty of Medicine, The University of Hong Kong, Hong Kong, Hong Kong

**Keywords:** Attention deficit hyperactivity disorder, Pharmacological treatment, Stimulants, Persistence, Adulthood

## Abstract

**Background:**

ADHD guidelines in the UK suggest that children and adults who respond to pharmacological treatment should continue for as long as remains clinically effective, subject to regular review. To what extent patients persist with treatment from childhood and adolescence into adulthood is not clear. This study aims to describe, in UK primary care, the persistence of pharmacological treatment for patients with ADHD who started treatment aged 6–17 years and to estimate the percentage of patients who continued treatment from childhood and adolescence into adulthood.

**Methods:**

The Health Improvement Network (THIN) database was used to identify patients with ADHD who received their first prescription for methylphenidate/ dexamfetamine/atomoxetine, aged 6–17 years. Patients were monitored until their ‘censored date’ (the earliest of the following dates: date the last prescription coded in the database ended, end of the study period (31st December 2008), date at which they transferred out of their practice, date of death, the last date the practice contributed data to the database). Persistence of treatment into adulthood was estimated using Kaplan Meier analysis.

**Results:**

610 patients had follow-up data into adulthood. 213 patients (93.4% male) started treatment between 6–12 years; median treatment duration 5.9 years. 131 (61.5%) stopped before 18 years, 82 (38.5%) were still on treatment age ≥18 years. 397 patients (86.4% male) started treatment between 13–17 years; median treatment duration was 1.6 years. 227 (57.2%) stopped before 18 years, 170 (42.8%) were still on treatment age ≥18 years. The number of females in both age categories was too small to formally test for differences between genders in persistence of treatment.

**Conclusion:**

Persistence of treatment into adulthood is lower (~40%) compared with published rates of persistence of the condition (~65% when symptomatic definition of remission used). Due to the limited number of patients with data past 18 years, it is important that ongoing monitoring of prescribing into later adulthood is undertaken, particularly to observe the effects of recommendations in new guidelines.

## Background

Attention deficit hyperactivity disorder (ADHD) was once perceived as a condition of childhood only; however increasing evidence has highlighted the existence of ADHD in adolescents and adults [1]. There is now consensus amongst experts that ADHD, diagnosed mainly using the broader Diagnostic and Statistical Manual of Mental Disorders 4th Edition (DSM-IV) criteria, may currently affect at least 1-2% of adults in the United Kingdom (UK) and 2-4% worldwide [2-6]. The prevalence of ADHD in school-aged children and adolescents in the UK using DSM-IV criteria is estimated at 5% [7]. The prevalence of hyperkinetic disorder (HKD) in school-aged children in the UK, characterised by the persistent traits of severe and pervasive inattentiveness, overactivity, and impulsiveness as defined by the International Classification of Diseases 10th Revision (ICD-10) criteria [8], is estimated at 1.5% [9].

Compared to children and adolescents, adults with ADHD are more likely to exhibit inattentive symptoms, as overt hyperactive symptoms tend to diminish with age [10]. However, they continue to suffer from symptoms such as the inability to sustain attention over a long period of time, disorganisation, forgetfulness and poor time management skills. Adolescents and adults with ADHD have higher rates of speeding offences and involvement in road traffic accidents than controls [10,11], are at an increased risk of substance use disorders (alcohol and drugs) [12] and becoming involved in crime and entering the criminal justice system [13]. Rates of separation and divorce have been reported to be higher in adults with ADHD [10,11].

There are two populations of adults with ADHD; those who are recognised and diagnosed by a health professional as having ADHD-associated impairments for the first time in adulthood, of whom some may have been previously misdiagnosed with other mental health conditions, and those who had a diagnosis of ADHD in childhood with symptoms and impairments persisting into later life. With regards to the latter cohort, studies conducted in this area have reported various rates of persistence of the disorder; a meta-analysis of follow-up studies in patients with ADHD [14] found that persistence was low (approximately 15% at age 25) when the syndromatic definition of remission was used (failing to meet the full diagnostic criteria for ADHD or the maintenance of full diagnostic status). However, the use of the symptomatic definition of remission (fewer than the number of symptoms required for a subthreshold diagnosis (i.e. fewer than five symptoms, or 36% of symptoms) or the maintenance of partial diagnostic status with impairment) resulted in a persistence rate of approximately 65% [14,15]. Importantly, under current plans for revision of the DSM criteria, it is proposed that many of the symptomatic remission cases will be re-classified as meeting full diagnostic criteria due to the persistence of four or more ADHD symptoms in either the inattentive or hyperactive-impulsive domains, associated with continued impairment [16].

While ADHD symptoms may persist in the majority of cases, many young people with ADHD make a good adjustment during young adulthood and do not require ongoing pharmacotherapy. In some cases however, long-term treatment may be warranted due to the persistence of impairing levels of ADHD symptoms. The National Institute for Health and Clinical Excellence (NICE), in the UK, recommend that if a child or adult responds adequately to treatment, then drug treatment for ADHD should be continued for as long as it remains clinically effective, with a review conducted at least on an annual basis [1].

Currently, in the UK, only the stimulant Concerta XL ® (prolonged-release methylphenidate) and the non-stimulant atomoxetine are indicated as continuation treatment in adults who started their treatment with this medication in childhood [17,18], however at the time the study was conducted, atomoxetine was the only licensed product for this indication.

Guidelines from North America [19], Europe [3] and the UK [1,20] advocate that appropriate treatments should be provided for adults with ADHD. Guidelines from the UK in particular [1,20] are expected to have significant effects on the prescribing of ADHD pharmacological treatments to adults in the UK; therefore an up-to-date study to investigate treatment patterns in adults with ADHD has been undertaken.

There are limited data on the treatment patterns of ADHD during the transition from childhood to adulthood in routine clinical practice. A recent longitudinal analysis in the UK of a cohort of 44 patients aged 15 years in 1999 demonstrated that 20% of patients were still receiving treatment at 18 years but none of the patients continued to receive treatment for ADHD beyond the age of 21 years; the research concluded that pharmacological treatment of some patients might have been stopped prematurely [21]. However this study investigated patients aged 15–21 years and only had data available up to December 2006 [21].

The Multimodal Treatment Study of Children with ADHD (MTA) was a multisite study in the US designed to evaluate the leading treatments for ADHD, including medication management, intensive behavioural treatment, combination treatment or community care (which included medication for approximately two-thirds of the sample). The children were followed up and results of the MTA study have been reported at 14, 24 and 36 months. At 14 months [22], the point at which randomisation ended, the outcome strongly favoured medication (whether or not in combination with behaviour therapy). Naturalistic follow-up at 24 [23] and 36 months [24], reported that treatment-related improvements for the children were generally maintained, but differential treatment efficacy was lost at and beyond 36-months [24].

The aim of the study is to estimate the persistence of pharmacological treatment for ADHD into adulthood for patients who started their treatment in childhood, or in adolescence, and to estimate the percentage of children and adolescents, stratified by gender, who continued long-term treatment for ADHD into adulthood, in the UK primary care setting.

## Methods

### Data source

A descriptive population-based cohort study was conducted using data from The Health Improvement Network (THIN). THIN contains anonymised computerised information entered by general practitioners (GPs) in the UK. With coverage of approximately 5.7% of the UK population (2009), practices that use the database are broadly representative of practices in the UK for patients’ characteristics [25]. GPs participating in THIN are trained to record information using the Vision software (In Practice Systems; London, UK) and the validity of the database for research has been supported by a number of studies [26-28]. Data recorded in THIN include patient demographics e.g. age, sex, details from GPs visits, diagnoses from specialist referrals and hospital admissions, and the results of laboratory tests. Prescriptions issued by the GP are directly generated from the computer. The Read Clinical Classification is used to code specific diagnoses and related signs and symptoms, and a drug dictionary based on data from the Multilex classification is used to code drugs. Prescriptions issued by specialists are not coded directly in the database but information on some of them may be available as free-text comments. Ethical approval was granted by the Cambridgeshire 4 Research Ethics Committee (ref: 09/H0305/81).

### Data extraction

The procedure for patient identification has been described previously in a study examining the prevalence and incidence of ADHD drug prescribing in children and adults in the UK [29]. In summary, patients were initially identified according to whether they had a prescription record for methylphenidate, dexamfetamine or atomoxetine (drugs approved by Medicines and Healthcare products Regulatory Agency (MHRA) in the UK for the treatment of ADHD) coded within the database between the study period of January 1st 2003 and 31st December 2008. All patients were required to have at least 12 months of continuous registration in the database.

Patients who had a diagnosis of ADHD/HKD coded within the database and had their first prescription for a study drug coded between the age of 6 and 17 years were identified and were classified as receiving their first prescription for a study drug either as a child (6–12 years) or as an adolescent (13–17 years). Age of individuals on the THIN database is calculated from the month and year of birth up to the age of 15 years. Once individuals reach the age of 15, age is calculated using the year of birth only (i.e. 1st January of that year).

The ‘index date’ for each patient was the date of their first prescription. [For some patients, this date preceded the start of the study period January 1st 2003, however these patients were eligible for inclusion if they remained on treatment during the study period]. Data were analysed from the index date to their ‘censored date’ which was the earliest of (i) date the last prescription coded ended (ii) end of the study period (31st December 2008) (iii) date at which they transferred out of their practice if this occurred before the end of the study period (iv) date of death (v) the last date the practice contributed data to the database. The duration of treatment was calculated for each patient. As an important aspect of this study was to determine persistence of treatment into adulthood (≥age 18 years), only patients who had continuous registration in the database up to and including age 18 years were included.

**Figure 1:**
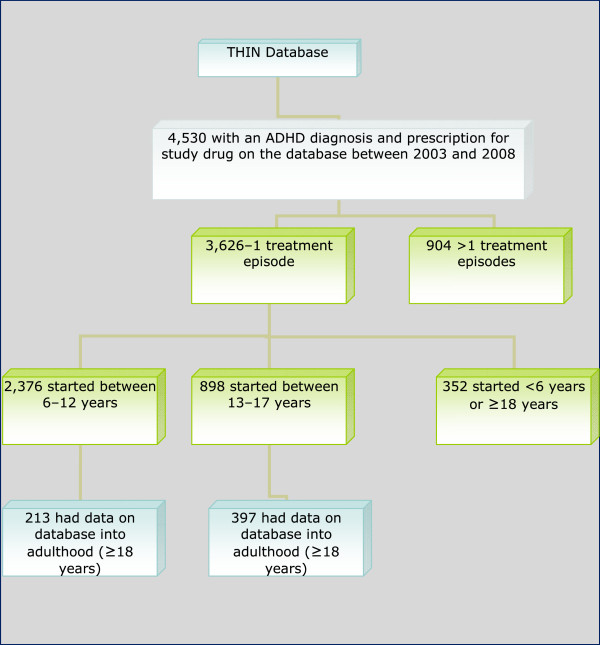
Flow chart of the process used to identify study participants.

The process used to identify these patients is presented in Figure 1.

### Data analysis

The duration of each prescription was calculated using the dose and quantity prescribed data. Based on previous work conducted on ADHD within the UK setting [21], an interval of more than 6 months between successive prescriptions was classified as treatment cessation. In certain cases, patients had more than one treatment episode i.e. patients received a prescription for a study drug more than 6 months after the end of the previous prescription. Patients with multiple treatment episodes were excluded from the current analyses as they were not considered persistent. The overall duration of treatment, by gender, was calculated from the start date of the first prescription to the calculated end date (i.e. date prescription was issued plus the duration of the prescription) of the last prescription coded in the database. Data manipulation and analyses were conducted using Stata/SE version 11.0 (StataCorp, College Station, Texas, United States).

### Outcomes

• To estimate the probability of continuing treatment for patients who started treatment in childhood and for those who started in adolescence, stratified by gender, using Kaplan-Meier analysis.

• To estimate the percentage of children and adolescents who persisted with ADHD pharmacological treatments into adulthood (≥18 years).

## Results

Over the six year study period, a total of 3274 patients between the age of 6 and 17 years met the criteria of initiating pharmacotherapy with one of the defined study drugs and one treatment episode. Over 80% (2664) of these patients did not have follow-up data beyond their 18th birthday; therefore they were not included in further analyses. However, 610 patients (18.6%) did have follow-up data in the database into adulthood (i.e. ≥18 years) and thus were included in the persistency calculations; 213 (34.9%; 199 male, 93.4%) patients started treatment between the age of 6 and 12 years and 397 (65.1%; 343 male, 86.4%) patients started between 13 and 17 years old.

For the 610 patients who met the study inclusion criteria, age at their ‘censored date’ [earliest of (i) date the last prescription coded ended (ii) end of the study period (31st December 2008) (iii) date at which they transferred out of their practice if this occurred before the end of the study period (iv) date of death (v) the last date the practice contributed data to the database] was calculated (Table 1).

**Table 1:** Age at ‘censored date’ for patients

**‘Censored Age’ (Years)**	**Number of patients 6**–**12 years (n=213)**	**Number of patients 13**–**17 years (n=397)**
**18**	**89**	**146**
**19**	**56**	**100**
**20**	**41**	**69**
**21**	**20**	**43**
**22**	**4**	**23**
**23**	**3**	**8**
**24**	**-**	**4**
**25**	**-**	**1**
**26**	**-**	**1**
**27**	**-**	**1**
**28**	**-**	**1**

### Patients who started treatment aged between 6 and 12 years

Table 2 presents data on the duration of treatment for the overall sample and stratified according to gender.

**Table 2:** Duration of treatment for patients who started treatment between 6 and 12 years of age

		**Median duration**	**Minimum duration**	**Maximum duration**	**Interquartile range**
Overall (n=213)		5.9 years	40 days	13.1 years	3.6 years
Males (n=199)		5.9 years	40 days	12.4 years	3.5 years
Females (n=14)		6.3 years	1.4 years	13.1 years	5.1 years

**Figure 2:**
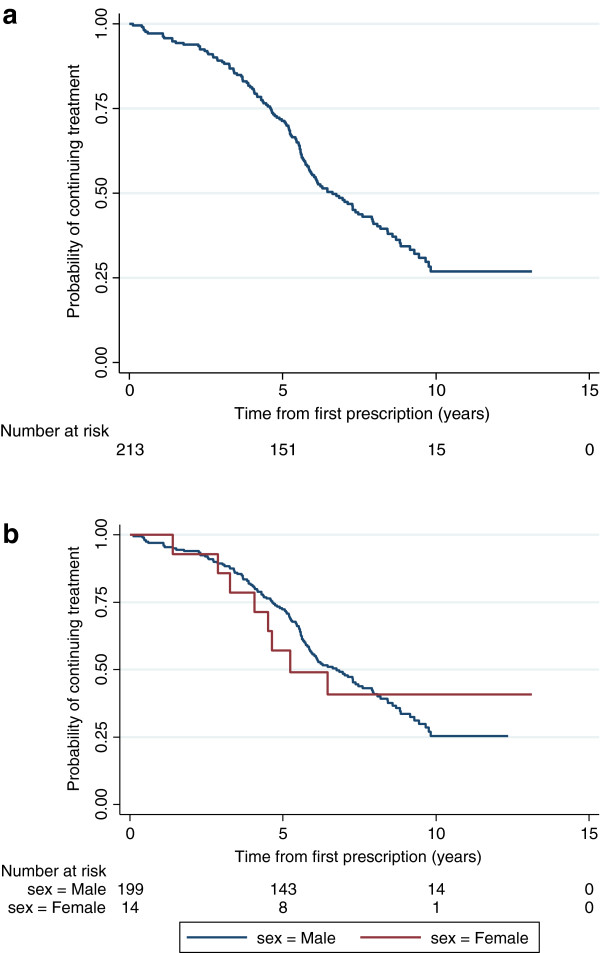
**a: Kaplan-Meier estimate of the probability of continuing pharmacological treatment for patients who started treatment between 6 and 12 years (n=213). b:** Kaplan-Meier estimate of the probability of continuing pharmacological treatment for male and female patients who started treatment between 6 and 12 years (n=213).

A Kaplan-Meier analysis was performed on the 213 patients who started treatment aged between 6 and 12 years. These data are presented in Figure 2a and stratified by gender in Figure 2b.

Of the 213 patients:

131 (61.5%) stopped treatment before age 18 years (123 male, 8 female)

82 (38.5%) were still on treatment at age ≥18 years (76 male, 6 female)

Of the 82 patients who were still on treatment at age ≥18 years, 15 patients (15 male; 18.3% of 82) subsequently stopped treatment within the study period. Thirteen of these patients stopped at age 18 years, one patient stopped at age 20 years, and one at age 22 years.

### Patients who started treatment aged between 13 and 17 years

Table 3 presents data on the duration of treatment for the overall sample and stratified according to gender.

**Table 3:** Duration of treatment for patients who started treatment between 13 and 17 years of age

	**Median duration**	**Minimum duration**	**Maximum duration**	**Interquartile range**
Overall (n=397)	1.6 years	7 days	12.1 years	2.4 years
Males (n=343)	1.6 years	7 days	12.1 years	2.4 years
Females (n=54)	1.6 years	7 days	6.6 years	2.7 years

**Figure 3:**
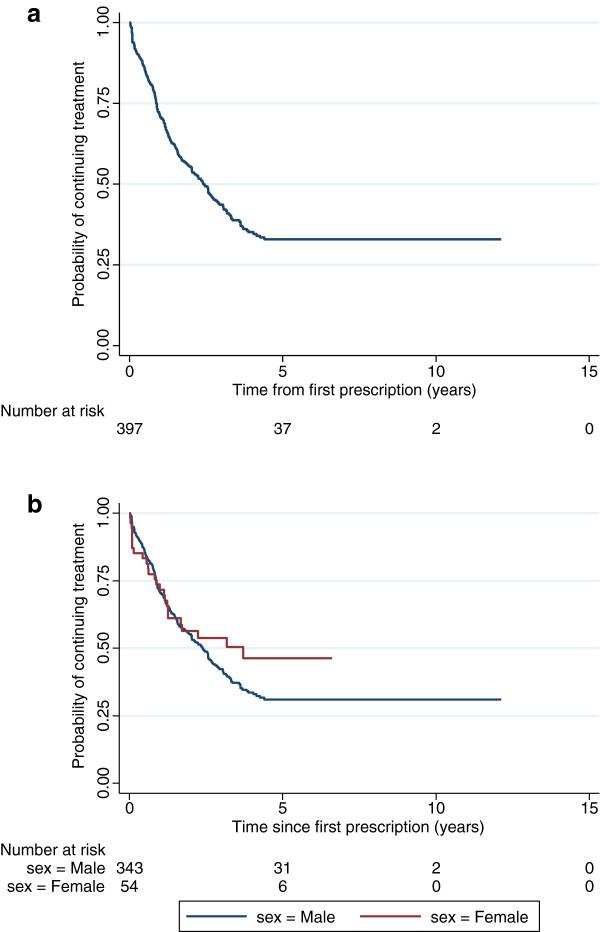
**a: Kaplan-Meier estimate of the probability of continuing pharmacological treatment for patients who started treatment between 13 and 17 years (n=397). b:** Kaplan-Meier estimate of the probability of continuing pharmacological treatment for male and female patients who started treatment between 13 and 17 years (n=397).

A second Kaplan-Meier analysis was performed on the 397 patients who started treatment aged between 13 and 17 years. These data for the overall sample are presented in Figure 3a and stratified by gender in Figure 3b.

Of the 397 patients:

227 (57.2%) stopped treatment before age 18 years (202 male, 25 female)

170 (42.8%) were still on treatment at age ≥18 years (141 male, 29 female)

Of the 170 patients, who were still on treatment at age ≥18 years, 45 (39 male, 6 female; 26.5% of 170) subsequently stopped treatment within the study period. Of these 45 patients who subsequently stopped treatment; 30 patients (26 male) stopped aged 18 years, nine patients (eight male) stopped at age 19 years, three patients (two male) stopped aged 20 years, one patient (male) stopped at aged 22 years, one patient (male) stopped aged 23 years, and one patient (male) stopped aged 24 years.

The records of the 358 patients who stopped treatment (131 who started treatment aged 6–12 years; 227 who started treatment aged 13–17 years) prior to reaching age 18 years were examined to ensure that these patients remained under the care of the GP and hadn’t left the practice. Of these 358 patients, 351 patients (98%) received at least one prescription (for any treatment) and/or had one medical record entered in the database after the calculated end date of their ADHD drug treatment.

## Discussion

It is well documented in follow-up studies as well as from reports of epidemiological surveys that impairments exist in people with ADHD that persist into adulthood [5,30]. What is less well known is to what extent patients persist with pharmacological treatment during the transition from childhood and adolescence into adulthood.

Pharmacological treatment patterns in our study cohort were examined to determine how many patients started treatment in childhood or adolescence and were continuing to have prescriptions issued by their GP up to and after turning 18 years. Two hundred and thirteen patients who started treatment between the age of 6 and 12 years had reached 18 years or older within the study period; of these 38.5% (82/213) were still receiving prescriptions for a study drug at 18 years of age. Of these 82 patients, 15 patients subsequently stopped within 4 years of turning 18.

Patients who started treatment in adolescence were also analysed to determine persistence into adulthood. Of these 397 patients, 43% (170/397) were still prescribed a study drug at age 18 years. Subsequently 45 patients stopped treatment aged ≥18 years.

It is recognised that once patients reach late adolescence, many factors are involved in determining whether a patient will remain on treatment. Some patients will find they have an improvement in symptoms and no longer require pharmacological treatment. Adolescents may choose not to continue treatment and this increase in self-autonomy has been reported in studies of adolescents with chronic diseases [31]. Adolescents may also decide to stop pharmacological treatment as there may be less expectation from parents and teachers for continued treatment as patients get older. Poor provision of treatment services for older adolescents and young adults may also contribute to the lower level of prescribing to these patients. Typically in the UK, patients with ADHD are under the care of child and adolescent mental health services up to the age of 16 years or school-leaving age with GPs responsible for prescribing ADHD medications [32]. However, ADHD services within adult mental health are currently poorly developed [33] and clear arrangements for transition are often lacking [34]. This can result in patients, in whom treatment for ADHD is clinically warranted, not having access to care by adult services for continuation of overall management [35]. Furthermore, the restricted licensing of the study drugs in adults may result in GPs being unwilling to prescribe pharmacological treatment in adulthood.

As expected, the majority of the sample was male. Higher ratios of males to females are observed in ADHD in children; however in adulthood the ratio appears to be equal [36]. The probability of persistence in females who started drug therapy at a younger age appeared to be lower than that of males, however the trend was reversed in patients starting in adolescence. Given the low number of female patients, we were unable to make any inferences on the differences observed between the genders.

A recent systematic review conducted by Shaw et al [37] examined the long-term outcomes of ADHD. Overall, the results showed that the long-term outcomes of ADHD when left untreated are poor compared with non-ADHD controls, and that treatment of ADHD improves long-term outcomes, but usually not to the level observed in non-ADHD individuals. These data reinforce the importance of treatment continuation for patients with ADHD as they transition from childhood and adolescence to adulthood.

### Strengths and limitations

A significant strength of the study was the use of a large database such as THIN which provided primary care data on a study cohort of over 4500 patients. THIN has been used widely in epidemiological research, including studies on mental health and in particular ADHD drug prescribing [29,38,39]. The use of longitudinal data allowed us to monitor the prescribing patterns to patients as they transition from childhood/adolescence to adulthood. However, the use of THIN primary care data does not capture all prescribing because some regions in the UK still depend on specialist services to prescribe medications for ADHD in children, adolescents or adults and these would not be systematically represented in the data from the THIN database.

A limitation of this study was that only a low proportion of patients reached 18 years within the study period (9% for patients starting in childhood, 44% for patients starting in adolescence). In addition, only 46 patients (7 patients starting in childhood, 39 patients starting in adolescence) had data in the database post 21 years of age, which limited analysis of persistence further into adulthood. The low number of patients also prevented analyses of differences in persistence between male and female patients. This is an area which warrants further investigation in the future. The persistence of ADHD past childhood/adolescence and its existence as a condition in adulthood has only been recognised in recent years and therefore continued surveillance using data from databases such as THIN is required to examine prescribing patterns further. Detailed information on the ADHD diagnoses is not systematically coded in the database and therefore it was not possible to determine the severity of ADHD, which has been demonstrated to predict persistence of ADHD from childhood into adulthood [40]. In addition, this research did not examine the presence of comorbid mental health conditions and the influence that these may have on the persistence of treatment. A follow-up questionnaire study that examines some of the issues behind treatment cessation and treatment persistence, including comorbid conditions was conducted on a sample of these patients and is reported separately.

#### Future work

In relation to persistence of prescribing of ADHD pharmacological treatment, a number of areas should be examined in the future, including the influence of psychosocial and comorbid factors; the influence of stimulant versus non-stimulant prescribing and the diagnostic stability of ADHD over the period from childhood to adulthood. While some of these data may be retrieved from databases such as THIN, they may require supplemental data from questionnaires to GPs or psychiatrists.

## Conclusion

Approximately 40% of patients who started pharmacological treatment for ADHD in childhood or adolescence continued to receive prescriptions at age 18 years. Although persistence into adulthood, as determined by GP prescribing, is higher than previously reported for the UK, figures are lower when compared to rates of persistence of the condition in adulthood. It is important that ongoing monitoring of GP prescribing patterns into later adulthood is undertaken, in particular to observe the effects of guideline recommendations such as those from NICE and the British Association for Psychopharmacology.

## Competing interests

LW and MM declare that they have no competing interests. SM received research funding as a result of involvement in this research study. PH is an employee and stock holder of Shire Pharmaceuticals Inc. Shire Pharmaceuticals develops and markets drugs to treat ADHD. PA has acted in an advisory role for Shire, Janssen-Cilag, Eli Lilly and Flynn Pharma. He has received education or research grants from Shire, Janssen-Cilag and Eli-Lilly. He has given talks at educational events sponsored by the above companies. ICKW was a member of National Institute for Health and Clinical Excellence (NICE) ADHD Guideline Group. ICKW has received funding from various pharmaceutical companies and NIHR; however, none of this funding is related to this study. ICKW has acted in an advisory role for Shire and Janssen-Cilag.

## Authors’ contributions

SM carried out the data analysis, interpretation and drafted the manuscript. LW contributed to the data analysis and interpretation; assisted with drafting the manuscript; reviewed the final manuscript. MM contributed to the study design, initial data analysis and review of the manuscript. PH conceived the need for the study, participated in the study design and development of the manuscript. PA contributed to initiating the project, conceiving the study and study questions and review and write up of the manuscript. ICKW contributed to initiating the study, conceiving the study and study questions, and also review and write-up of the manuscript. All authors read and approved the final manuscript.

## Pre-publication history

The pre-publication history for this paper can be accessed here:

http://www.biomedcentral.com/1471-244X/12/219/prepub

## References

[B1] Attention deficit hyperactivity disorderpharmacological and psychological interventions in children, young people and adults http://www.nice.org.uk/CG72

[B2] Rosler M Casas M Konofal E Buitelaar J Attention deficit hyperactivity disorder in adults World J Biol Psychiatry 2010 11 5 684 698 20521876 10.3109/15622975.2010.483249

[B3] Kooij SJ Bejerot S Blackwell A Caci H Casas-Brugue M Carpentier PJ Edvinsson D Fayyad J Foeken K Fitzgerald M European consensus statement on diagnosis and treatment of adult ADHD: The European Network Adult ADHD BMC Psychiatry 2010 10 67 20815868 10.1186/1471-244X-10-67 PMC2942810

[B4] Asherson P Clinical assessment and treatment of attention deficit hyperactivity disorder in adults Expert Rev Neurother 2005 5 4 525 539 16026236 10.1586/14737175.5.4.525

[B5] Fayyad J De Graaf R Kessler R Alonso J Angermeyer M Demyttenaere K De Girolamo G Haro JM Karam EG Lara C Cross-national prevalence and correlates of adult attention-deficit hyperactivity disorder Br J Psychiatry 2007 190 402 409 17470954 10.1192/bjp.bp.106.034389

[B6] Simon V Czobor P Balint S Meszaros A Bitter I Prevalence and correlates of adult attention-deficit hyperactivity disorder: meta-analysis Br J Psychiatry 2009 194 3 204 211 19252145 10.1192/bjp.bp.107.048827

[B7] Methylphenidateatomoxetine and dexamfetamine for attention deficit hyperactivity disorder (ADHD) in children and adolescents Technology Appraisal 98 http://www.nice.org.uk/TA98

[B8] World Health Organisation International classification of diseases, 10th revision: the classification of mental and behavioural disorders: clinical descriptions and diagnostic guidelines 1992 Geneva: WHO

[B9] Mental health of children and young people in Great Britain 2004 http://www.dh.gov.uk/en/Publicationsandstatistics/Publications/PublicationsStatistics/DH_4118332

[B10] Faraone SV Biederman J Spencer T Wilens T Seidman LJ Mick E Doyle AE Attention-deficit/hyperactivity disorder in adults: an overview Biol Psychiatry 2000 48 1 9 20 10913503 10.1016/s0006-3223(00)00889-1

[B11] Wilens TE Faraone SV Biederman J Attention-deficit/hyperactivity disorder in adults JAMA 2004 292 5 619 623 15292088 10.1001/jama.292.5.619

[B12] Gudjonsson GH Sigurdsson JF Sigfusdottir ID Young S An epidemiological study of ADHD symptoms among young persons and the relationship with cigarette smoking, alcohol consumption and illicit drug use J Child Psychol Psychiatry 2011 53 3 304 312 22066497 10.1111/j.1469-7610.2011.02489.x

[B13] Young SJ Adamou M Bolea B Gudjonsson G Muller U Pitts M Thome J Asherson P The identification and management of ADHD offenders within the criminal justice system: a consensus statement from the UK Adult ADHD Network and criminal justice agencies BMC Psychiatry 2011 11 32 21332994 10.1186/1471-244X-11-32 PMC3050801

[B14] Faraone SV Biederman J Mick E The age-dependent decline of attention deficit hyperactivity disorder: a meta-analysis of follow-up studies Psychol Med 2006 36 2 159 165 16420712 10.1017/S003329170500471X

[B15] Biederman J Mick E Faraone SV Age-dependent decline of symptoms of attention deficit hyperactivity disorder: impact of remission definition and symptom type Am J Psychiatry 2000 157 5 816 818 10784477 10.1176/appi.ajp.157.5.816

[B16] Diagnostic and Statistical Manual of Mental Disorders (DSM-5) Developmenthttp://www.dsm5.org 10.1590/s2317-17822013000200017 24413388

[B17] Janssen-Cilag Ltd Concerta XL. Summary of Product Characteristics Electronic Medicines Compendium Available from URL: http://www.medicines.org.uk/EMC/medicine/19549/SPC/Concerta; [updated 13/09/2011] Accessed: 15th November 2011

[B18] Eli Lilly and Company Limited Atomoxetine (Strattera) Summary of Product Characteristics Electronic Medicines Compendium Available from http://www.medicines.org.uk/EMC/medicine/14482/SPC/Strattera; [updated 08/12/2011] Accessed: 15th June 2011

[B19] Canadian Attention Deficit Hyperactivity Disorder Resource Alliance (CADDRA) Canadian ADHD Practice Guidelines 2011 Third Toronto ON

[B20] Nutt DJ Fone K Asherson P Bramble D Hill P Matthews K Morris KA Santosh P Sonuga-Barke E Taylor E Evidence-based guidelines for management of attention-deficit/hyperactivity disorder in adolescents in transition to adult services and in adults: recommendations from the British Association for Psychopharmacology J Psychopharmacol 2007 21 1 10 41 17092962 10.1177/0269881106073219

[B21] McCarthy S Asherson P Coghill D Hollis C Murray M Potts L Sayal K de Soysa R Taylor E Williams T Attention-deficit hyperactivity disorder: treatment discontinuation in adolescents and young adults Br J Psychiatry 2009 194 3 273 277 19252159 10.1192/bjp.bp.107.045245

[B22] The MTA Cooperative Group A 14-month randomized clinical trial of treatment strategies for attention-deficit/hyperactivity disorder. Multimodal Treatment Study of Children with ADHD Arch Gen Psychiatry 1999 56 12 1073 1086 10591283 10.1001/archpsyc.56.12.1073

[B23] National Institute of Mental Health Multimodal Treatment Study of ADHD follow-up 24-month outcomes of treatment strategies for attention-deficit/hyperactivity disorder Pediatrics 2004 113 4 754 761 15060224 10.1542/peds.113.4.754

[B24] Jensen PS Arnold LE Swanson JM Vitiello B Abikoff HB Greenhill LL Hechtman L Hinshaw SP Pelham WE Wells KC 3-year follow-up of the NIMH MTA study J Am Acad Child Adolesc Psychiatry 2007 46 8 989 1002 17667478 10.1097/CHI.0b013e3180686d48

[B25] McBride D Hardoon S Walters K Gilmour S Raine R Explaining variation in referral from primary to secondary care: cohort study BMJ 2010 341 c6267 21118873 10.1136/bmj.c6267 PMC2995017

[B26] Haynes K Forde KA Schinnar R Wong P Strom BL Lewis JD Cancer incidence in The Health Improvement Network Pharmacoepidemiol Drug Saf 2009 18 8 730 736 19479713 10.1002/pds.1774

[B27] Lo Re V 3rd Haynes K Forde KA Localio AR Schinnar R Lewis JD Validity of The Health Improvement Network (THIN) for epidemiologic studies of hepatitis C virus infection Pharmacoepidemiol Drug Saf 2009 18 9 807 814 19551699 10.1002/pds.1784 PMC2735008

[B28] Lewis JD Schinnar R Bilker WB Wang X Strom BL Validation studies of the health improvement network (THIN) database for pharmacoepidemiology research Pharmacoepidemiol Drug Saf 2007 16 4 393 401 17066486 10.1002/pds.1335

[B29] McCarthy S Wilton L Murray ML Hodgkins P Asherson P Wong ICK The epidemiology of pharmacologically-treated attention deficit hyperactivity disorder (ADHD) in children, adolescents and adults in UK primary care BMC Pediatrics 2012 12 78 22712630 10.1186/1471-2431-12-78 PMC3472167

[B30] Kessler RC Adler L Barkley R Biederman J Conners CK Demler O Faraone SV Greenhill LL Howes MJ Secnik K The prevalence and correlates of adult ADHD in the United States: results from the National Comorbidity Survey Replication Am J Psychiatry 2006 163 4 716 723 16585449 10.1176/appi.ajp.163.4.716 PMC2859678

[B31] Miller VA Drotar D Decision-making competence and adherence to treatment in adolescents with diabetes J Pediatr Psychol 2007 32 2 178 188 16717139 10.1093/jpepsy/jsj122

[B32] National Service Framework for Children Young People and Maternity Services. Transition: Getting it Right for Young People UK: Improving the Transition of Young People with Long-Term Conditions from Children’s to Adult Health Services http://www.dh.gov.uk/assetRoot/04/13/21/49/04132149.pdf

[B33] Asherson P Chen W Craddock B Taylor E Adult attention-deficit hyperactivity disorder: recognition and treatment in general adult psychiatry Br J Psychiatry 2007 190 4 5 17197649 10.1192/bjp.bp.106.026484

[B34] ADHD - Services Over Scotland. Report of the Service Profiling Exercisehttp://www.healthcareimprovementscotland.org/programmes/mental_health/adhd/adhd_service_profile.aspx

[B35] Wong IC Asherson P Bilbow A Clifford S Coghill D DeSoysa R Hollis C McCarthy S Murray M Planner C Cessation of attention deficit hyperactivity disorder drugs in the young (CADDY)--a pharmacoepidemiological and qualitative study Health Technol Assess 2009 13 50 1 120 iii-iv, ix-xi 10.3310/hta13490 19883527

[B36] Kooij JJ Buitelaar JK van den Oord EJ Furer JW Rijnders CA Hodiamont PP Internal and external validity of attention-deficit hyperactivity disorder in a population-based sample of adults Psychol Med 2005 35 6 817 827 15997602 10.1017/s003329170400337x

[B37] Shaw M Hodgkins P Caci H Young S Kahle J Woods AG Arnold LE A systematic review and analysis of long-term outcomes in attention deficit hyperactivity disorder: effects of treatment and non-treatment BMC Med 2012 10 1 99 22947230 10.1186/1741-7015-10-99 PMC3520745

[B38] Morgan O Griffiths C Majeed A Antidepressant prescribing and changes in antidepressant poisoning mortality and suicide in England, 1993–2004 J Public Health (Oxf) 2008 30 1 60 68 18239187 10.1093/pubmed/fdm085

[B39] Martin-Merino E Ruigomez A Wallander MA Johansson S Garcia-Rodriguez LA Prevalence, incidence, morbidity and treatment patterns in a cohort of patients diagnosed with anxiety in UK primary care Fam Pract 2010 27 1 9 16 19884124 10.1093/fampra/cmp071

[B40] Kessler R Adler L Barkley R Biederman J Conners CK Faraone S Greenhill L Jaeger S Secnik K Spencer T Patterns and predictors of ADHD persistence into adulthood: Results from the National Comorbidity Survey Replication Biol Psychiatry 2005 57 11 1442 1451 15950019 10.1016/j.biopsych.2005.04.001 PMC2847347

